# P-1418. The Use of Whole Genome Sequencing and a Novel Bioinformatic Pipeline for Mycobacterium abscessus Molecular Epidemiology

**DOI:** 10.1093/ofid/ofaf695.1605

**Published:** 2026-01-11

**Authors:** Xavier Quan-Nguyen, Maxime Veillette, Nicholas Waglechner, Pier-Alexandre Vasil, Floriane Point, Bouchra Tannir, Melissa Zarandi-Nowroozi, Catherine Tsimiklis, Nadine Pétrin, Marty Steven Teltscher, Anna Urbanek, Pierre-Marie Akochy, Joseph Cox, Robyn Lee, Simon Grandjean Lapierre

**Affiliations:** Université de Montréal, Montréal, Canada, Montréal, QC, Canada; Université de Montréal, Montréal, Canada, Montréal, QC, Canada; Mount Sinai Hospital, Toronto, Canada, Toronto, Ontario, Canada; Université de Montréal, Montréal, Canada, Montréal, QC, Canada; Centre de Recherche du Centre Hospitalier de l’Université de Montréal, Montréal, Canada, Montréal, Quebec, Canada; Université de Montréal, Montréal, Canada, Montréal, QC, Canada; Université de Montréal, Montréal, Canada, Montréal, QC, Canada; Hôpital du Sacré-Coeur de Montréal, Montréal, Canada, Montréal, Quebec, Canada; Centre Hospitalier de l'Université de Montréal, Montréal, Canada, Montréal, Quebec, Canada; Jewish General Hospital, Montréal, Canada, Montréal, Quebec, Canada; Direction régionale de santé publique, CIUSSS du Centre-Sud-de-l’île-de-Montréal, Montréal, Canada, Montréal, Quebec, Canada; Institut National de Santé Publique du Québec, Montréal, Canada, Montréal, Quebec, Canada; Faculty of Medicine and Health Sciences, McGill University, Montréal, Canada, Montréal, Quebec, Canada; Dalla Lana School of Public Health, University of Toronto, Toronto, Canada, Montréal, Quebec, Canada; Centre de Recherche du Centre Hospitalier de l’Université de Montréal, Montréal, Canada, Montréal, Quebec, Canada

## Abstract

**Background:**

Bacterial genotyping can support outbreak investigations including skin and soft tissue infections point source outbreaks caused by *Mycobacterium abscessus*. This study presents an innovative approach that combines Nanopore long-read and Illumina short-read whole-genome sequencing (WGS) data using a novel bioinformatic pipeline to identify related *M. abscessus* isolates.

**Figure:**
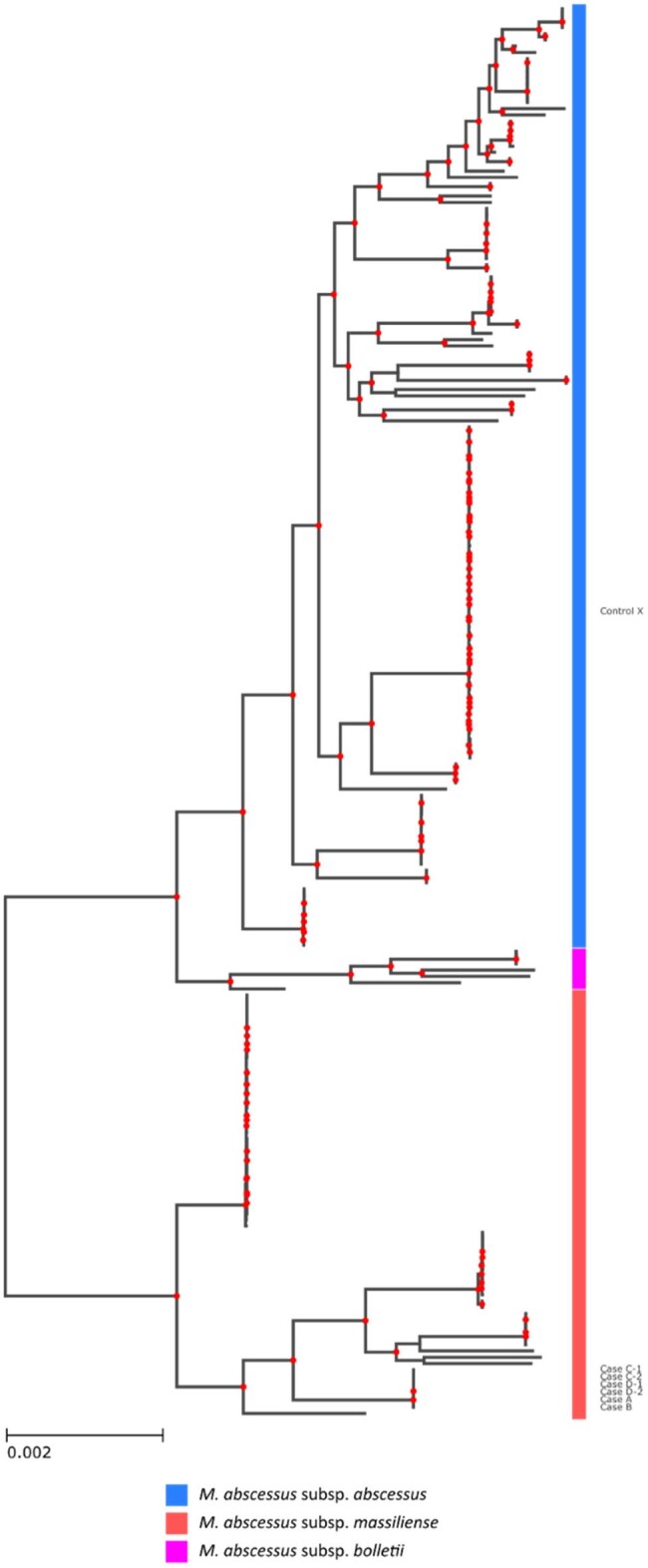
Core-genome SNP phylogeny of Montreal M. abscessus isolates The core genome was estimated from open reading frames of the assembled outbreak isolates along with 220 additional isolates from the island of Montreal. M. abscessus subsp. massiliense isolates cultured from the skin infection outbreak patients exhibit high relatedness compared to the Montreal M. abscessus catalog.

**Methods:**

Physicians and public health authorities identified a potential skin and soft tissue infection outbreak in Montreal, Canada. Six isolates from four patients were retrieved and DNA was sequenced using both Illumina short-read and Oxford Nanopore long-read WGS platforms.Minhash *k*-mer distances to reference *M. abscessus* type strains were used to confirm taxonomic identity of clinical isolates. Complete genomes for each isolate were assembled using long-reads and polished using short-read data. Single nucleotide polymorphism (SNP) distances between clinical isolates and the putative earliest outbreak case were used to examine their relatedness. Outbreak isolates were also compared to 220 contemporary Montreal *M. abscessus* genomes using core-genome (cg) SNPs to assess their relatedness and clustering among locally circulating strains.

**Results:**

All outbreak isolates were most closely related to *M. abscessus* subsp. *massilliense*. Short reads from the outbreak isolates showed 100% mapping coverage to the complete assembly of the earliest outbreak isolate. All outbreak isolates were found to be nearly identical, differing by only 0-2 SNPs. In comparison, using the more distantly related subspecies reference exaggerated SNP distances between isolates (32-50 SNPs), and between outbreak isolates and the reference (26612-26644 SNPs). The cgSNP phylogenetic tree shows the outbreak isolates are distinct to other Montreal *M. abscessus* genomes and to the *M. abscessus* subspecies reference genomes.

**Conclusion:**

Our findings indicate that this improved molecular approach leveraging both short and long read sequence data to produce complete genome assemblies of *M. abscessus.* This helped confirm the relatedness of epidemiologically linked cases and assess their bacterial isolates’ molecular clustering within an extended catalog of locally circulating strains.

**Disclosures:**

All Authors: No reported disclosures

